# Real-World Effectiveness of Palbociclib Plus Letrozole vs Letrozole Alone for Metastatic Breast Cancer With Lung or Liver Metastases: Flatiron Database Analysis

**DOI:** 10.3389/fonc.2022.865292

**Published:** 2022-07-04

**Authors:** Adam Brufsky, Xianchen Liu, Benjamin Li, Lynn McRoy, Rachel M. Layman

**Affiliations:** ^1^ University of Pittsburgh Medical Center Hillman Cancer Center, University of Pittsburgh Medical Center (UPMC), School of Medicine, Pittsburgh, PA, United States; ^2^ Medical Pfizer Inc, New York, NY, United States; ^3^ Department of Breast Medical Oncology The University of Texas MD Anderson Cancer Center, Houston, TX, United States

**Keywords:** HR+/HER2−, metastatic breast cancer, palbociclib, real-world data, visceral metastasis

## Abstract

**Background:**

Cyclin-dependent kinase 4/6 inhibitors are a standard treatment for patients with hormone receptor−positive (HR+)/human epidermal growth factor receptor 2−negative (HER2−) metastatic breast cancer (MBC). However, real-world data on effectiveness in patients with liver or lung metastatic disease is limited. This study compared outcomes of palbociclib plus letrozole versus letrozole alone in patients with HR+/HER2− MBC with lung or liver metastasis treated in routine clinical practice in the United States.

**Methods:**

This retrospective analysis used Flatiron Health’s database of electronic health records. Women with HR+/HER2− MBC and liver or lung metastasis received first-line palbociclib plus letrozole or letrozole alone between February 2015 and February 2019. Real-world progression-free survival (rwPFS) was defined as time from start of treatment to death or disease progression. Stabilized inverse probability treatment weighting (sIPTW) was used to balance baseline demographic and clinical characteristics between palbociclib plus letrozole versus letrozole cohorts. Cox proportional-hazards models were used to estimate the effectiveness of palbociclib plus letrozole versus letrozole alone in rwPFS and overall survival (OS).

**Results:**

The study included 353 patients with lung metastasis, 123 with liver metastasis, and 75 with both. After sIPTW, palbociclib plus letrozole versus letrozole alone was significantlly associated with prolonged rwPFS (hazard ratio (HR), 0.56) and OS (HR, 0.58) (both p<0.001) in all patients. Palbociclib plus letrozole compared with letrozole alone demonstrated a median rwPFS of 16.5 versus 10.5 months, respectively (adjusted HR, 0.52; *P*<0.001), a median OS of not reached versus 40.3 months (adjusted HR, 0.60; *P*<0.01) in patients with lung metastasis, and median OS of 30.1 versus 16.8 months (adjusted HR, 0.56; *P*<0.03 in patients with liver metastasis. In patients with liver metastasis, palbociclib plus letrozole had a median rwPFS of 10.7 months versus 8.0 months in the letrozole alone cohort (adjusted HR, 0.70; *P*=0.12).

**Conclusions:**

In this real-world population, palbociclib in combination with letrozole is associated with improved outcomes compared with letrozole alone for patients with HR+/HER2− MBC and liver or lung metastasis in the first-line setting. The findings support first-line palbociclib in combination with an aromatase inhibitor as standard of care for HR+/HER2− MBC regardless of visceral disease.

**Clinical Trial Registration:**

NCT04176354.

## Introduction

Breast cancer is the most common malignancy in women, accounting for more than 43,000 deaths in the United States in 2021 (1). Approximately 6% of patients with breast cancer are initially diagnosed with metastatic disease and, of patients who are diagnosed with early-stage disease, about 30% will go on to develop metastatic breast cancer (MBC). Despite improvements in treatment, MBC has a poor prognosis and an overall 5-year survival rate of only 27% for patients in the United States (2).

Hormone receptor-positive (HR+) breast cancer, which accounts for approximately 70% of all breast cancers, most commonly metastasizes to the bone, lung, and liver, with <10% metastasizing to the brain and other tissues (3, 4). An analysis of more than 9500 patients with HR+/human epidermal growth factor receptor 2−negative (HER2−) MBC found that patients with brain and liver metastasis had significantly lower overall survival (OS) compared with those with lung metastasis, whereas patients with bone metastasis exhibited the most favorable OS (3).

Endocrine-based therapy (ET) is recommended as a first-line treatment for patients with HR+ advanced breast cancer (5). A recent meta-analysis of patients with HR+/HER2− advanced breast cancer receiving ET (either an aromatase inhibitor, fulvestrant, or tamoxifen) found that progression-free survival (PFS) and OS were significantly longer in patients with non-visceral versus visceral metastatic disease (6). Furthermore, patients with liver metastasis had a significantly shorter PFS and OS than patients with non-liver visceral metastasis. Similarly, in a single-center study of patients with HR+/HER2− MBC receiving fulvestrant, PFS was nearly the same in patients with non-visceral metastasis and lung metastasis (without liver metastasis) whereas those with liver metastasis had significantly worse PFS (7). These findings demonstrate the heterogeneity in MBC with respect to response to therapy and highlight the difficulty in treating patients with liver metastasis.

Cyclin-dependent kinase 4/6 inhibitors (CDK4/6i) in combination with ET have become the standard of care for HR+/HER2− MBC (8). The safety and efficacy of palbociclib, the first-in-class CDK4/6i inhibitor, in combination with letrozole as a first-line treatment for patients with estrogen receptor–positive/HER2− MBC were demonstrated in the phase 2 PALOMA-1 trial and subsequently validated in the phase 3 PALOMA-2 trial (9, 10). A subgroup analysis of patients in PALOMA-2 demonstrated that palbociclib in combination with letrozole provided a significant PFS benefit compared with letrozole in combination with placebo in those with visceral and non-visceral disease (10). A recent retrospective analysis of a real-world population of patients with HR+/HER2− MBC with visceral crisis found that patients receiving CDK4/6i had a 5-month improvement in OS compared with those receiving chemotherapy, consistent with CDK4/6i providing benefit in patients with MBC with visceral disease (11).

Observational real-world studies complement findings from clinical trials, providing important evidence demonstrating a therapy’s efficacy in populations that are more heterogeneous than those in clinical trials (12). An understanding of real-world treatment practices and how they affect the efficacy of new therapies can also help guide clinicians on optimal drug use and indications (13). In this study, we evaluated the efficacy of palbociclib in combination with letrozole as a first-line treatment in patients with HR+/HER2− MBC with lung or liver metastasis in a real-world setting.

## Materials and Methods

### Database

This was a retrospective analysis of electronic health records (EHRs) from the Flatiron Health’s analytic longitudinal database. The Flatiron database undergoes rigorous data curation and abstraction and is regarded as one of the industry’s foremost oncology databases (14). It includes de-identified structured and unstructured EHRs from more than 280 cancer clinics, including approximately 800 sites of care, and represents 2.4 million patients with cancer actively being treated in the United States. This database has been validated and has been widely used for multiple real-world studies in cancer, including breast cancer (15–17).

### Patients

Patients included in the analysis were female and ≥18 years old, were diagnosed with HR+/HER2− MBC with liver or lung involvement, and initiated palbociclib plus letrozole or letrozole alone in the first-line setting between February 2015 and February 2019. The study data cutoff was May 31, 2019, to allow a potential minimum follow-up of 3 months from the date of palbociclib plus letrozole or letrozole initiation (ie, index date). Patients were excluded if they had previously been treated with a CDK4/6i, an aromatase inhibitor, tamoxifen, raloxifene, toremifene, or fulvestrant for MBC or with a CDK4/6i as part of a clinical trial. Only patients whose first structured activity was ≤90 days after their MBC diagnosis date were included to exclude patients who might have been treated for their MBC. Patients were followed up from the start of treatment with palbociclib plus letrozole or letrozole alone to death, the last visit, or study end, whichever came first. This retrospective deidentified database analysis was exempt from institutional review board approval and included a waiver of informed consent.

### Outcomes

Real-world PFS (rwPFS) was defined as the time from the start of treatment to death or disease progression, whichever came first, as described previously (18). Disease progression was determined by the recorded assessment of the treating clinician based on radiology, laboratory evidence, pathology, or clinical evaluation. Patients who did not die or experience disease progression were censored at the date of initiation of the next line of therapy (for patients with ≥2 lines of therapy) or their last visit during the study period (for patients with only 1 line of therapy). OS was defined as the length of time from the start of the first line of therapy to the date of death due to any cause, as previously described (18). The date of death was determined by Flatiron based on a recent mortality dataset generated by combining multiple data sources and benchmarked against the National Death Index. If a patient did not die during the study period, they were censored at the end of study.

### Statistical Analyses

Descriptive statistics were used for demographic and clinical characteristics. Follow-up duration was defined as the time from the start of treatment with palbociclib plus letrozole or letrozole alone to death, the last visit, or study end, whichever came first.

Stabilized inverse probability treatment weighting (sIPTW) and propensity score matching (PSM) methods were used to balance baseline demographic and clinical characteristics between comparison cohorts (palbociclib plus letrozole vs letrozole alone), as previously described (18). PSM was conducted as a sensitivity analysis to assess the robustness of the sIPTW results. Propensity scores were generated using a multivariable binomial logistic regression model with age group, number of metastatic sites, practice type, race, disease stage, Eastern Cooperative Oncology Group (ECOG) performance status, and cancer type (*de novo* vs recurrent) as covariates (19). Matches were made using 1:1 nearest neighbor matching without replacement.

The Kaplan-Meier method was used to estimate medians and 95% CIs and for landmark analyses for rwPFS and OS. To compare rwPFS and OS between treatment groups, univariate and multivariate Cox proportional hazards models with a robust sandwich estimator were used for patients with lung and liver metastasis, respectively. In multivariate analyses, models included age group, number of metastatic sites, practice type, race, disease stage, ECOG performance status, and cancer type (*de novo* vs recurrent).

Chi-squared test was performed to compare disease progression during the second-line treatment in patients who were initially treated with palbociclib plus letrozole versus letrozole alone.

## Results

### Patients

A total of 551 eligible patients, 353 (64.1%) with lung metastasis, 123 (22.3%) with liver metastasis, and 75 (13.6%) with both lung and liver metastases, were included in the analysis. Of these patients, 330 (59.9%) initiated first-line therapy with palbociclib plus letrozole and 221 (40.1%) with letrozole alone. The median (interquartile range) follow-up was 22.6 (17.3) and 22.1 (21.5) months for patients receiving palbociclib plus letrozole and letrozole, respectively. Demographic and clinical characteristics are shown in **Table 1**. Across both treatment groups, approximately 95% of patients were from a community setting and about 4.0% from an academic setting. Compared with the letrozole group, patients in the palbociclib plus letrozole group tended to be younger, were less likely to be Black, and were more likely to have a better ECOG performance status and ≥3 metastatic sites. A small percentage of patients, 4.2% in the palbociclib plus letrozole group and 6.3% in the letrozole group, also had brain metastases. Patient characteristics were generally balanced after sIPTW and propensity score matching (**Table 1**).

**Table 1:** Patient demographic and clinical characteristics.

	Unadjusted			sIPTW			PSM		
Characteristic	Palbociclib + letrozole(n=330)	Letrozole(n=221)	Standardized difference	Palbociclib + letrozole(n=321)	Letrozole(n=269)	Standardized difference	Palbociclib + letrozole(n=194)	Letrozole(n=194)	Standardized difference
Age, y									
Mean (SD)	65.7 (10.2)	70.0 (10.6)	–0.410	67.0 (10.4)	67.7 (11.5)	–0.063	66.7 (11.0)	68.9 (10.6)	–0.201
Median (IQR)	66.0 (15.0)	71.0 (18.0)		67.0 (15.0)	68.0 (17.0)		67.0 (17.0)	69.0 (17.0)	
18−49	24 (7.3)	10 (4.5)	0.117	20 (6.3)	14 (5.1)	0.049	16 (8.2)	10 (5.2)	0.124
50−64	121 (36.7)	56 (25.3)	0.247	106 (32.9)	90 (33.4)	–0.010	60 (30.9)	54 (27.8)	0.068
65−74	115 (34.8)	64 (29.0)	0.127	106 (33.0)	85 (31.7)	0.027	63 (32.5)	61 (31.4)	0.022
75+	70 (21.2)	91 (41.2)	–0.441	89 (27.8)	80 (29.7)	–0.042	55 (28.4)	69 (35.6)	–0.155
Race/ethnicity*									
White	219 (66.4)	146 (66.1)	0.006	212 (66.0)	178 (66.2)	–0.003	106 (54.6)	131 (67.5)	–0.267
Black	20 (6.1)	26 (11.8)	–0.201	21 (6.4)	26 (9.7)	–0.122	20 (10.3)	16 (8.2)	0.071
Asian	3 (0.9)	5 (2.3)	–0.109	3 (1.0)	5 (1.9)	–0.080	3 (1.5)	4 (2.1)	–0.039
Hispanic or Latino	9 (2.7)	3 (1.4)	0.097	8 (2.5)	6 (2.0)	0.032	4 (2.1)	3 (1.5)	0.039
Other/Unknown	79 (23.9)	41 (18.6)	0.132	77 (24.0)	54 (20.1)	0.095	61 (31.4)	40 (20.6)	0.249
Practice type*									
Academic	17 (5.2)	8 (3.6)	0.075	15 (4.8)	10 (3.7)	0.052	8 (4.1)	7 (3.6)	0.027
Community	313 (94.8)	213 (96.4)		305 (95.2)	259 (96.3)		186 (95.9)	187 (96.4)	
Insurance type									
Commercial Health Plan + any other	69 (20.9)	55 (24.9)	–0.095	73 (22.6)	61 (22.7)	–0.001	32 (24.1)	34 (25.6)	–0.035
Commercial Health Plan	85 (25.8)	37 (16.7)	0.222	77 (23.7)	50 (18.5)	0.127	31 (23.3)	26 (19.5)	0.092
Medicare	12 (3.6)	13 (5.9)	–0.106	16 (5.0)	13 (4.9)	0.005	5 (3.8)	4 (3.0)	0.042
Medicaid	4 (1.2)	2 (0.9)	0.030	4 (1.2)	3 (1.1)	0.017	1 (0.8)	1 (0.8)	0
Other payer type	160 (48.5)	114 (51.6)	–0.062	153 (47.4)	142 (52.8)	–0.109	64 (48.1)	68 (51.1)	–0.060
Disease stage at diagnosis*									
I	38 (11.5)	22 (10.0)	0.050	38 (11.7)	24 (8.8)	0.097	25 (12.9)	21 (10.8)	0.064
II	86 (26.1)	42 (19.0)	0.170	79 (24.8)	56 (20.9)	0.093	37 (19.1)	39 (20.1)	–0.026
III	42 (12.7)	26 (11.8)	0.029	40 (12.4)	34 (12.6)	–0.005	27 (13.9)	23 (11.9)	0.062
IV	122 (37.0)	90 (40.7)	–0.077	118 (36.7)	112 (41.6)	–0.102	70 (31.6)	78 (40.2)	–0.085
Not documented	42 (12.7)	41 (18.6)	–0.161	46 (14.4)	43 (16.0)	–0.047	35 (18.0)	33 (17.0)	0.027
ECOG performance status*									
0	126 (38.2)	54 (24.4)	0.300	105 (32.8)	89 (33.1)	–0.007	57 (29.4)	54 (27.8)	0.034
1	70 (21.2)	45 (20.4)	0.021	68 (21.2)	55 (20.6)	0.015	44 (22.7)	41 (21.1)	0.037
2, 3, or 4	18 (5.5)	34 (15.4)	–0.329	25 (7.9)	30 (11.3)	–0.113	18 (9.3)	22 (11.3)	–0.068
Not documented	116 (35.2)	88 (39.8)	–0.097	122 (38.1)	94 (35.0)	0.064	75 (38.7)	77 (39.7)	–0.021
Brain metastases	14 (4.2)	14 (6.3)	–0.094	13 (4.1)	22 (8.2)	–0.170	10 (5.2)	14 (7.2)	–0.086
Time from initial diagnosis to metastatic diagnosis,* y									
* De novo*	122 (37.0)	90 (40.7)	–0.077	118 (36.7)	112 (41.6)	–0.102	70 (36.1)	78 (40.2)	–0.085
≤1	7 (2.1)	4 (1.8)	0.022	7 (2.0)	6 (2.1)	–0.002	4 (2.1)	4 (2.1)	0
>1–≤5	47 (14.2)	25 (11.3)	0.088	47 (14.7)	30 (11.3)	0.101	24 (12.4)	23 (11.9)	0.016
>5	154 (46.7)	102 (46.2)	0.010	149 (46.5)	121 (45.0)	0.032	96 (49.5)	89 (45.9)	0.072
Number of metastatic sites*^,†^									
1	66 (20.0)	51 (23.1)	–0.075	74 (23.0)	46 (17.1)	0.147	43 (22.2)	37 (19.1)	0.077
2	119 (36.1)	88 (39.8)	–0.078	117 (36.6)	107 (39.8)	–0.067	82 (42.3)	81 (41.8)	0.010
3	91 (27.6)	56 (25.3)	0.051	83 (25.7)	76 (28.4)	–0.061	43 (22.2)	52 (26.8)	–0.108
4	36 (10.9)	17 (7.7)	0.111	30 (9.5)	25 (9.4)	0.005	17 (8.8)	15 (7.7)	0.038
≥5	18 (5.5)	9 (4.1)	0.065	17 (5.2)	14 (5.2)	–0.003	9 (4.6)	9 (4.6)	0
Duration of follow-up, mo									
Mean (SD)	23.6 (12.3)	21.9 (14.3)	0.124	23.5 (12.3)	21.6 (15.7)	0.133	23.5 (12.4)	21.5 (14.5)	0.145
Median (IQR)	22.6 (17.3)	22.1 (21.5)		22.6 (17.7)	22.1 (21.2)		22.3 (17.7)	20.6 (22.6)	

All data are n (%) unless otherwise noted.

The balance in prognostic baseline characteristics was determined using a standardized difference approach, with a standardized difference of ≥0.10, considered indicative of practical significance (19). The total patient population for the different subgroups varied owing to the use of sIPTW. Therefore, the total number for each subgroup may not have equaled the number in the treatment arm (owing to rounding errors or differences in categorization). Percentages were based on the number of patients reported within each subgroup.

ECOG, Eastern Cooperative Oncology Group; IQR, interquartile range; PSM, propensity score matching; sIPTW, stabilized inverse probability treatment weighting.

*Variable used in the propensity score matching model; de novo vs not de novo were used as categories for initial diagnosis to metastatic diagnosis.

^†^Multiple metastasis at the same site were counted as 1 site.

### rwPFS and OS in Patients With Lung or Liver Metastasis

Evaluation of landmark rwPFS demonstrated that the percentage of patients with rwPFS at 6 months was higher in the palbociclib plus letrozole group (76.2%) versus the letrozole group (63.2%), and this benefit was maintained at 24 months (38.5% vs 22.4%).

**Figure 1:**
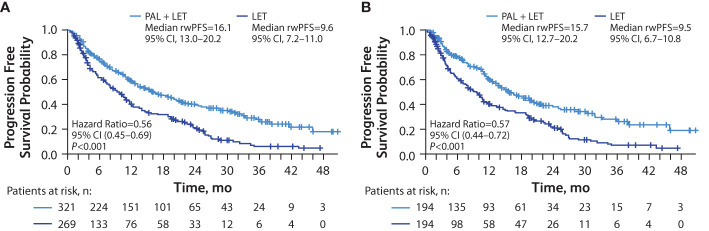
Kaplan-Meier curves of real-world progression-free survival in patients with visceral (lung and/or liver) metastasis. sIPTW analysis **(A)** and PSM analysis **(B)**; number of patients at risk are shown. LET, letrozole; PAL, palbociclib; PSM, propensity score matching; rwPFS, real-world progression-free survival; sIPTW, stabilized inverse probability treatment weighting.

In the unadjusted analysis of patients with visceral metastasis, median rwPFS was significantly longer among those in the palbociclib plus letrozole group versus the letrozole group (15.4 [95% CI, 12.5–19.5] months vs 10.2 [95% CI, 8.0–11.7] months; hazard ratio, 0.60 [95% CI, 0.49–0.74]; *P*<0.001). In the sIPTW-adjusted analysis of patients with visceral metastasis, median rwPFS was significantly longer among those in the palbociclib plus letrozole group versus the letrozole group (16.1 [95% CI, 13.0–20.2] months vs 9.6 [95% CI, 7.2–11.0] months; hazard ratio, 0.56 [95% CI, 0.45–0.69]; *P*<0.001; **Figure 1A**). In a sensitivity analysis using the PSM method, median rwPFS was also significantly longer among those in the palbociclib plus letrozole group versus the letrozole group (15.7 [95% CI, 12.7–20.2] months vs 9.5 [95% CI, 6.7–10.8] months; hazard ratio, 0.57 [95% CI, 0.44–0.72]; *P*<0.001; **Figure 1B**)

The percentage of patients with OS at 12 months was higher in the palbociclib plus letrozole group (89.4%) versus the letrozole group (75.0%), and this benefit was maintained at 36 months (59.7% vs 44.5%).

**Figure 2:**
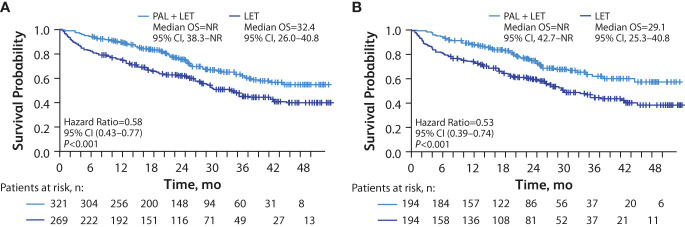
Kaplan-Meier curves of overall survival in patients with visceral (lung and/or liver) metastasis. sIPTW analysis **(A)** and PSM analysis **(B)**; number of patients at risk are shown. LET, letrozole; NR, not reached; OS, overall survival; PAL, palbociclib; PSM, propensity score matching; sIPTW, stabilized inverse probability treatment weighting.

Among the patients with visceral metastasis, the median OS was significantly longer in the palbociclib plus letrozole group versus the letrozole group in the unadjusted analysis (not reached [NR; 95% CI, 38.3–NR] months vs 29.4 [95% CI, 25.8–40.8] months; hazard ratio, 0.56 [95% CI, 0.43–0.74]; *P*<0.001) and in the sIPTW-adjusted analysis (NR [95% CI, 38.3–NR] months vs 32.4 [95% CI, 26.0–40.8] months; hazard ratio, 0.58 [95% CI, 0.43–0.77]; *P*<0.001; **Figure 2A**). In a sensitivity analysis using the PSM method, median OS was also significantly longer among those in the palbociclib plus letrozole group versus the letrozole group (NR [95% CI, 42.7–NR] months vs 29.1 [95% CI, 25.3–40.8] months; hazard ratio, 0.53 [95% CI, 0.39–0.74]; *P*<0.001; **Figure 2B**)

### Outcomes in Patients With Lung Metastasis

**Figure 3:**
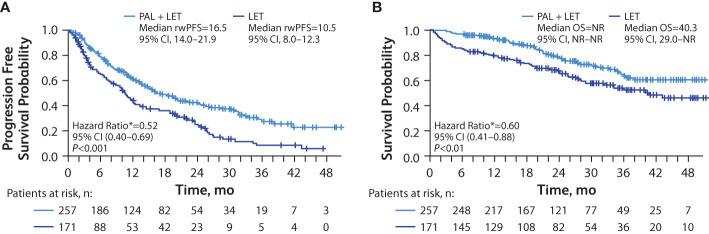
Kaplan-Meier curves of real-world progression-free survival and overall survival in patients with lung metastasis. Analysis of rwPFS **(A)** and OS **(B)** adjusted for demographic and clinical variables; number of patients at risk are shown. LET, letrozole; NR, not reached; OS, overall survival; PAL, palbociclib; rwPFS, real-world progression-free survival. *Hazard ratios were estimated by multivariate Cox regression models adjusted for baseline demographic and clinical variables.

In patients with lung metastasis, median rwPFS was significantly longer among those in the palbociclib plus letrozole group versus the letrozole group in unadjusted analysis (16.5 [95% CI, 14.0–21.9] months vs 10.5 [95% CI, 8.0–12.3] months; hazard ratio, 0.58 [95% CI, 0.45–0.74]; *P*<0.001) and remained significantly longer among those in the palbociclib plus letrozole group versus the letrozole group after adjusting for baseline covariates hazard ratio, 0.52 [95% CI, 0.40–0.69]; *P*<0.001; **Figure 3A**). Median OS was also significantly longer among those in the palbociclib plus letrozole group versus the letrozole group in unadjusted analysis (NR [95% CI, NR–NR] vs 40.3 [95% CI, 29.0–NR] months; hazard ratio, 0.58 [95% CI, 0.41–0.82]; *P*<0.01 and in the multivariate analysis hazard ratio, 0.60 [95% CI, 0.41–0.88]; *P*<0.01; **Figure 3B**).

### Outcomes in Patients With Liver Metastasis

**Figure 4:**
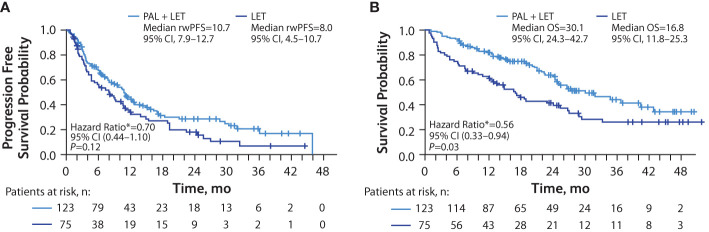
Kaplan-Meier curves of real-world progression-free survival and overall survival in patients with liver metastasis. Analysis of rwPFS **(A)** and OS **(B)** adjusted for demographic and clinical variables; number of patients at risk are shown. LET, letrozole; OS, overall survival; PAL, palbociclib; rwPFS, real-world progression-free survival. *Hazard ratios were estimated by multivariate Cox regression models adjusted for baseline demographic and clinical variables.

In patients with liver metastasis, median rwPFS was significantly longer among those in the palbociclib plus letrozole group versus the letrozole group in unadjusted analysis (10.7 [95% CI, 7.9–12.7] months vs 8.0 [95% CI, 4.5–10.7] months; hazard ratio, 0.71 [95% CI, 0.51–0.99]; *P*=0.04). After adjusting for baseline covariates, the median rwPFS remained longer among those in the palbociclib plus letrozole group versus the letrozole group hazard ratio, 0.70 [95% CI, 0.44–1.10]; *P*=0.12; **Figure 4A**), although the analysis did not reach statistical significance.

Median OS was significantly longer among those in the palbociclib plus letrozole group versus the letrozole group in the unadjusted analysis (30.1 [95% CI, 24.3–42.7] months vs 16.8 [95% CI, 11.8–25.3] months; hazard ratio, 0.55 [95% CI, 0.37–0.81]; *P*<0.01 and remained significantly longer among those in the palbociclib plus letrozole group versus the letrozole group in the multivariate analysis hazard ratio, 0.56 [95% CI, 0.33–0.94]; *P*=0.03; **Figure 4B**).

### Subsequent Second-Line Anticancer Treatments and Disease Progression During the Second-Line Treatment

**Table 2** shows treatments following first-line treatment and disease progression among patients receiving second-line treatment. Among patients with lung metastasis, 51.4% in the palbociclib plus letrozole group and 60.8% in the letrozole group had second line treatment. Of the patients in the palbociclib plus letrozole group, 36.4% and 28.0% received a CDK4/6i and chemotherapy, respectively, as a second-line treatment. Of the patients in the letrozole group, 60.6% and 13.5% received a CDK4/6i and chemotherapy, respectively, as a second-line treatment. Among the patients with lung metastasis that were being treated in the second line, there was no significant difference in the proportion of patients that experienced disease progression between the palbociclib plus letrozole group and the letrozole group (52.3% vs 45.2%, χ^2^ = 1.17; *P*=0.28).

**Table 2:** Subsequent second-line anticancer treatments and disease progression in the second-line treatment.

	Patients with liver metastasis		Patients with lung metastasis	
Treatments, n (%)	Palbociclib + letrozole (n=123)	Letrozole (n=75)	Palbociclib + letrozole (n=257)	Letrozole (n=171)
First-line treatment only*	49 (39.8)	28 (37.3)	125 (48.6)	67 (39.1)
Any second-line treatment received^†^	74 (60.2)	47 (62.7)	132 (51.4)	104 (60.8)
CDK4/6 inhibitor	22 (29.8)	23 (48.9)	48 (36.4)	63 (60.6)
Chemotherapy	26 (35.1)	6 (12.8)	37 (28.0)	14 (13.5)
Endocrine therapy alone	8 (10.8)	14 (29.8)	24 (18.2)	20 (19.2)
Other anticancer treatment	21 (28.4)	7 (14.9)	32 (24.2)	15 (14.4)
Second-line disease progression	37 (50.0)	20 (42.6)	69 (52.3)	47 (45.2)

CDK4/6=cyclin-dependent kinase 4/6.

*Includes patients who continued treatment, died, or were censored in the first-line setting.

^†^Patients could have received >1 category of second-line treatment.

Among patients with liver metastasis, 60.2% in the palbociclib plus letrozole group and 62.7% in the letrozole group had second-line treatment. Of the patients in the palbociclib plus letrozole group, 29.8% and 35.1% received a CDK4/6i and chemotherapy, respectively, as a second-line treatment. Of the patients in the letrozole group, 48.9% and 12.8% received a CDK4/6i and chemotherapy, respectively, as a second line treatment. Among the patients with liver metastasis that were being treated in the second line, there was no significant difference in disease progression between patients in the palbociclib plus letrozole group and the letrozole group (50.0% vs 42.6%, χ^2^ = 0.64; *P*=0.42).

## Discussion

Lung and liver are common sites of metastasis in HR+/HER2− MBC (4). Visceral involvement at these sites has been associated with poorer prognosis, and metastatic liver disease has been shown to be particularly difficult to treat (3, 6). Previous subgroup analysis of patients in the PALOMA-2 trial showed that first-line palbociclib in combination with letrozole provided a PFS benefit compared with letrozole alone in patients with visceral and nonvisceral disease (10). However, real-world data on the efficacy of CDK4/6i in patients with lung and liver metastasis are scarce. This study demonstrates in a real-world population that first-line palbociclib in combination with letrozole provided a significant benefit in rwPFS and OS compared with letrozole alone in patients with lung or liver metastasis after adjusting for important demographic and clinicopathologic factors.

Subgroup exploratory analyses in clinical trials have shown that a CDK4/6i prolongs PFS in patients with HR+/HER2− advanced breast cancer and visceral metastasis. The MONALEESA-7 and MONALEESA-2 trials of pre/perimenopausal and postmenopausal women, respectively, with HR+/HER2− advanced breast cancer and visceral metastasis have shown that ribociclib in combination with ET provided a significant PFS benefit compared with placebo in combination with ET (20, 21). A pooled analysis of 7 phase 3 trials in patients with HR+/HER2− advanced breast cancer receiving first-line CDK4/6i (N=1111) demonstrated that CDK4/6i plus ET provided greater PFS benefit compared with placebo plus ET in patients with visceral metastasis, with a hazard ratio similar to those in the broader intended-use population (22). In our real-world population of patients with visceral metastasis (lung and/or liver), first-line palbociclib in combination with letrozole significantly prolonged rwPFS compared with letrozole alone. When analysis was restricted to patients with lung metastasis, palbociclib in combination with letrozole also significantly prolonged rwPFS compared with letrozole alone.

Previous studies have also provided evidence that treatment with CDK4/6i prolongs OS in patients with visceral metastasis. A subgroup analysis of patients with pre/perimenopausal HR+/HER2− advanced breast cancer with liver or lung metastasis who enrolled in the MONALEESA-7 trial demonstrated that ribociclib in combination with ET provided an OS benefit compared with placebo plus ET, although the results did not reach statistical significance (23). A large meta-analysis of HR+/HER2− MBC patients from the MONALEESA-3 and -7, MONARCH 2 and PALOMA 3 trials (N=1390) demonstrated that CDK4/6i in combination with ET (first or laterr lines), compared with ET alone, significantly prolonged OS in subgroups of patients with and without visceral involvement (24). A recent retrospective analysis of a real-world population of patients with HR+/HER2− MBC evaluated OS in patients with visceral crisis at diagnosis (N=336) (11). Patients receiving CDK4/6i had a 5-month improvement in OS compared with those receiving chemotherapy (11). In this real-world study, first-line palbociclib in combination with letrozole also significantly prolonged OS in patients with visceral metastasis (lung and/or liver) and patients with lung metastasis compared with letrozole alone.

Compared with patients with HR+ MBC and visceral non-liver metastasis or lung metastasis, patients with liver metastasis have been shown to respond poorly to treatment with ET, underscoring the more aggressive course of disease in patients with liver involvement (6, 7). A recent subgroup analysis of patients with HR+/HER2− MBC and liver metastasis enrolled in the MONARCH trials demonstrated that abemaciclib in combination with ET as first-line treatment was shown to provide a substantial benefit over ET alone, characterized by significantly prolonged PFS (25). In our population of patients with liver metastasis, palbociclib in combination with letrozole was associated with a significant benefit over letrozole alone in rwPFS. After adjusting for covariates, the benefit of palbociclib in combination with letrozole over letrozole alone remained (hazard ratio = 0.70, palbociclib plus letrozole vs letrozole), although the results did not reach statistical significance. The small sample size of patients with liver metastases (n=198 [palbociclib + letrozole, n=123; letrozole alone, n=75]) makes this analysis difficult to interpret. However, in the OS analysis of patients with liver metastasis, the addition of palbociclib to letrozole significantly lengthened OS compared with letrozole alone, even after adjustment for covariates.

Our findings in this real-world population are consistent with findings from clinical trials, and demonstrate that palbociclib in combination with ET as first line treatment is effective in patients with more difficult to treat lung and liver metastatic disease. Recently, it was demonstrated that in a real-world population of HR+/HER2− advanced breast cancer patients receiving CDK4/6i plus ET in the first line, the proliferative index marker Ki67 was significantly inversely correlated with PFS, suggesting that this may be a marker of CDK4/6i resistance (26). In addition, a recent gene expression analysis of tumors from patients in two neoadjuvant trials of CDK4/6i plus ET found that tumors from patients that exhibited intrinsic resistance to CDK4/6i were highly enriched in interferon-related signatures (27). Such findings suggest that future clinical studies evaluating the Ki67 index and interferon signaling as biomarkers of CDK4/6i resistance are warranted.

By the study cutoff date, 40%-50% of patients remained on first-line treatment. Among patients who received second-line treatment, CDK4/6is were more commonly used in the letrozole alone cohort than in the palbociclib plus letrozole cohort. However, no significant difference in disease progression during second-line treatment was observed between patients who were initially treated with palbociclib plus letrozole versus letrozole alone. Further research with large sample sizes is warranted to demonstrate the effects of subsequent treatments following first-line CDK4/6i treatment on disease progression and OS.

Although the large size and geographic distribution of the Flatiron database is a strength of this study, there are inherent limitations to retrospective analysis of real-world data. The quality of information extracted from the EHR depends on the quality of information entered by the clinician, and there is a potential for missing or incomplete data.

Unobserved variables cannot be completely addressed through multivariate analysis; however, our analyses did adjust for known clinical confounders that were most likely to affect the outcomes of the study. Sample sizes for subgroup analyses, especially for patients with liver metastasis, are small for robust statistical tests. Also, for OS analysis, the median OS was reached in the letrozole alone group; although significant censoring in the OS analysis indicates the need for subsequent evaluation with longer follow-up. The greater generalizability of the findings may be limited because patients in the Flatiron database may not be reflective of the general population of patients with MBC. Also, rwPFS determination was not based on Response Evaluation Criteria in Solid Tumors (RECIST) criteria and was limited by each clinician’s interpretation and documentation of tumor responses. Furthermore, no causal relationship could be made from the retrpospective database analysis.

### Conclusions

This comparative analysis provides evidence that addition of palbociclib to letrozole as first-line therapy significantly improves outcomes for patients with HR+/HER2− MBC with lung or liver metastasis in routine clinical practice. These findings are consistent with subgroup analyses in patients with visceral metastasis from pivotal clinical trials and will better inform clinicians on appropriate therapeutic strategies for patients with poor prognostic characteristics, such as liver metastasis. Further comparative effectiveness research of CDK4/6i combined with ET involving more patients and longer follow-up is warranted in patients with MBC with various visceral metastases.

## Data Availability Statement

The data analyzed in this study are subject to the following licenses/restrictions: Upon request, and subject to review, Pfizer will provide the data that support the findings of this study. Subject to certain criteria, conditions and exceptions, Pfizer may also provide access to the related individual de-identified participant data. Requests to access these datasets should be directed to https://www.pfizer.com/science/clinical-trials/trial-data-and-results for more information.

## Ethics Statement

The study was retrospective, non-interventional, and used anonymized data provided by Flatiron Health. The Flatiron database is covered under the Health Insurance Portability and Accountability Act of 1996 (HIPAA) through Business Associate Agreements with every provider in the Flatiron network. Ethical approval was not provided for this study on human participants because this study is exempt from institutional review board approval and included a waiver of informed consent. Written informed consent for participation was not required for this study in accordance with the national legislation and the institutional requirements.

## Author Contributions

All authors listed have made a substantial, direct, and intellectual contribution to the work and approved it for publication.

## Funding

Pfizer Inc (NCT04176354).

## Conflict of Interest

AB, Consulting fees or honorarium from Pfizer Inc, AstraZeneca, Lilly, Novartis, and Sanofi. XL, BL, and LM, Pfizer employee and Pfizer stockholder. RL, Consulting fees from Pfizer Inc, Eli Lilly, Celcuity, and Novartis, research support from Pfizer Inc, Eli Lilly, Novartis, GlaxoSmithKline, Puma, Zentalis, and Celcuity.

The authors declare that this study received funding from Pfizer Inc. The funder had the following involvement with the study: data acquisition, study design, data analysis, and coauthoring the paper. No authors received funding or payment for coauthoring the paper from the funder.

## Publisher’s Note

All claims expressed in this article are solely those of the authors and do not necessarily represent those of their affiliated organizations, or those of the publisher, the editors and the reviewers. Any product that may be evaluated in this article, or claim that may be made by its manufacturer, is not guaranteed or endorsed by the publisher.
